# Individual Variation of the Genetic Response to Bisphenol A in Human Foreskin Fibroblast Cells Derived from Cryptorchidism and Hypospadias Patients

**DOI:** 10.1371/journal.pone.0052756

**Published:** 2012-12-28

**Authors:** Xian-Yang Qin, Hideko Sone, Yoshiyuki Kojima, Kentaro Mizuno, Katsuhiko Ueoka, Koji Muroya, Mami Miyado, Aya Hisada, Hiroko Zaha, Tomokazu Fukuda, Jun Yoshinaga, Junzo Yonemoto, Kenjiro Kohri, Yutaro Hayashi, Maki Fukami, Tsutomu Ogata

**Affiliations:** 1 Health Risk Research Section, Research Center for Environmental Risk, National Institute for Environmental Studies, Tsukuba, Ibaraki, Japan; 2 Department of Environmental Studies, Graduate School of Frontier Science, University of Tokyo, Kashiwa, Chiba, Japan; 3 Department of Urology, Fukushima Medical University School of Medicine, Fukushima, Fukushima, Japan; 4 Department of Nephro-Urology, Nagoya City University Graduate School of Medical Sciences, Nagoya, Aichi, Japan; 5 Department of Surgical Subspecialties, National Research Center for Child Health and Development, Tokyo, Japan; 6 Division of Endocrinology and Metabolism, Kanagawa Children's Medical Center, Kanagawa, Yokohama, Japan; 7 Department of Endocrinology and Metabolism, National Research Institute for Child Health and Development, Tokyo, Japan; 8 Department of Animal Production Science, Graduate School of Agricultural Science, Tohoku University, Sendai, Miyagi, Japan; 9 Department of Pediatrics, University Hospital, Hamamatsu University School of Medicine, Hamamatsu, Shizuoka, Japan; University Paris Diderot/University Paris 7, France

## Abstract

**Background/Purpose:**

We hypothesized that polymorphic differences among individuals might cause variations in the effect that environmental endocrine disruptors (EEDs) have on male genital malformations (MGMs). In this study, individual variation in the genetic response to low-dose bisphenol A (BPA) was investigated in human foreskin fibroblast cells (hFFCs) derived from child cryptorchidism (CO) and hypospadias (HS) patients.

**Methodology/Principal Findings:**

hFFCs were collected from control children without MGMs (*n* = 5) and child CO and HS patients (*n* = 8 and 21, respectively). BPA exposure (10 nM) was found to inhibit matrix metalloproteinase-11 (*MMP11*) expression in the HS group (0.74-fold, *P* = 0.0034) but not in the control group (0.93-fold, *P* = 0.84) and CO group (0.94-fold, *P* = 0.70). Significantly lower levels of *MMP11* expression were observed in the HS group compared with the control group (0.80-fold, *P* = 0.0088) and CO group (0.79-fold, *P* = 0.039) in response to 10 nM BPA. The effect of single-nucleotide polymorphism *rs5000770* (G>A), located within the aryl hydrocarbon receptor nuclear translocator 2 (*ARNT2*) locus, on individual sensitivity to low-dose BPA was investigated in the HS group. A significant difference in neurotensin receptor 1 (*NTSR1*) expression in response to 10 nM BPA was observed between AA and AG/GG groups (*n* = 6 and 15, respectively. *P* = 0.031). However, no significant difference in *ARNT2* expression was observed (*P* = 0.18).

**Conclusions/Significance:**

This study advances our understanding of the specificity of low-dose BPA effects on human reproductive health. Our results suggest that genetic variability among individuals affects susceptibility to the effects of EEDs exposure as a potential cause of HS.

## Introduction

Cryptorchidism (CO) and hypospadias (HS) are the two most common congenital male genital malformations (MGMs) with a global prevalence of approximately 2–9% and 0.2–1%, respectively [1], [2]. The etiologies of CO and HS are complicated and have been partly related with *in utero* exposure to environmental endocrine disruptors (EEDs) [3], [4], [5]. However, epidemiological studies on this issue have produced conflicting results [6]. It is believed that the effect of EEDs depend on several factors, including the dosage of EEDs exposure, the developmental stage during which EEDs exposure occurred, and genetic variability to the effects of EEDs exposure [7]. We have previously investigated the association between single-nucleotide polymorphisms (SNPs) of genes involved in EEDs metabolism and the risk of CO and HS in a Japanese population and found that SNP *rs5000770* (G>A) within intron 1 of aryl hydrocarbon receptor nuclear translocator 2 (*ARNT2*) was significantly associated at both allele and genotype levels with increased risk of CO and HS [8].

ARNT2 is a member of the basic helix-loop-helix Per-ARNT-SIM (bHLH-PAS) family of transcription factors that is involved in the regulation of many physiological pathways, including responses to environmental contaminants [9], [10]. *Arnt2* plays pivotal roles in the regulation of early development in zebrafish [11] and *ARNT2* knockout mice suffer severe developmental defects and die shortly after birth [12], [13]. *ARNT2* polymorphisms have been linked with the risk of some specific congenital malformations in humans such as cleft palate [14]. However, little is known about the relationship of *ARNT2* polymorphisms and the risk of MGMs. Therefore, we aimed to investigate whether the polymorphic differences among individuals might cause variations in the ability of EEDs to cause MGMs. It is likely that further investigations on this issue will shed increased light on the link between EEDs exposure and the development of MGMs.

Bisphenol A (BPA) is used extensively in the manufacture of the plastics used to make food and beverage containers and has a global production of over six billion pounds per year [15]. However BPA is a well-known estrogen-like EED. BPA has been detected in 92% of urine samples in a US reference population, suggesting that humans might be continuously exposed to this compound in their daily lives [16]. To better understand the molecular basis of the effect of low-dose BPA exposure on human reproductive health, we previously performed a genome-wide screen using human foreskin fibroblast cells (hFFCs) derived from child HS patients to identify novel targets of low-dose BPA exposure [17]. We reported that the expression of matrix metalloproteinase-11 (*MMP11*), a well-known effector of development and normal physiology, was downregulated by BPA in a dose-dependent manner. We also demonstrated that *MMP11* expression was significantly lower in the HS group compared with that in the CO group. These findings indicated that *MMP11* is an important target of low-dose BPA and the involvement of BPA in the development of HS might relate to downregulation of *MMP11* expression.

In this study, to better understand the effect of BPA exposure on human reproductive health, individual susceptibility to low-dose BPA was investigated in hFFCs derived from child CO and HS patients. Human foreskin tissues obtained from patients with HS have been used as *in vitro* models to define the etiology of HS [18], [19]. In addition to *MMP11*, neurotensin receptor 1 (*NTSR1*), known to be involved in tumor progression by regulating a series of transforming functions such as cellular migration and invasion, was also selected as a candidate BPA-responsive gene [20]. We did not identify *NTSR1* as a target of low-dose BPA in our previous genome-wide screen, probably owing to a lack of statistical significance [17]. However, this may indicate variability in gene expression levels among individuals in response to low-dose BPA exposure (Figure S1).

## Materials and Methods

### Sample collection

hFFCs were obtained from control children with concealed penis or phimosis and child HS and CO patients undergoing surgical procedures at the National Research Institute for Child Health and Development, Japan, during 2007–2009. With more details, foreskin specimens were obtained from children with HS and phimosis during HS repair and circumsicion, respectively. Then, the foreskin specimens were mechanically dissociated with scissors into small pieces and cultured on plastic plates. The adhered cells on the plate were then obtained as hFFCs. All subjects were of Japanese origin, and written informed consent was obtained from the guardians of each participant. This study was approved by the Institutional Ethics Committees of the Nagoya City University Graduate School of Medical Sciences, the National Research Institute for Child Health and Development and the National Institute for Environmental Studies.

### Chemicals

Dimethyl sulfoxide (DMSO) was obtained from Sigma Chemical Co. (St. Louis, MO, USA), and BPA was obtained from Wako Industries (Osaka, Japan). DMSO was used as the primary solvent, and the DMSO solutions were further diluted in cell culture media prior to use. The final concentration of DMSO in media did not exceed 0.1% (vol/vol).

### Cell culture

hFFCs were maintained in Dulbecco's modified Eagle's medium (DMEM)/Ham's F-12 (048-29785, Wako, Osaka, Japan) containing 10% fetal bovine serum (FBS; Mediatech, Herndon, VA, USA) and grown at 37°C in a 5% CO_2_ humidified incubator. For growth under steroid-free conditions, cells were seeded in phenol red-free DMEM/Ham's F-12 (045-30665, Wako) containing 5% charcoal/dextran-treated FBS (Hyclone, Logan, UT, USA). All culture media contained 100 U/ml penicillin/streptomycin and 2 mmol/L L-glutamine (Mediatech, Herndon, VA, USA).

### RNA isolation and reverse-transcription polymerase chain reaction (RT-PCR)

Total RNA was isolated from cultured cells after treatment with DMSO or BPA for 24 h using an RNeasy Kit (Qiagen, Valencia, CA, USA) in accordance with the manufacturer's instructions. Quantification and quality assessment of the isolated RNA were performed using an Agilent Bioanalyzer 2100 (Agilent Technologies, Palo Alto, CA, USA) and a NanoDrop spectrophotometer (NanoDrop products, Wilmington, DE, USA) in accordance with the manufacturer's instructions. Complementary DNA (cDNA) was synthesized using a High Capacity RNA-to-cDNA Kit (Applied Biosystems, Foster City, CA, USA) according to the manufacturer's instructions. The primers (Forward: 5′-ACCCTCCGGAAATGGCTTCAGA-3′; Reverse: 5′-CATCGGTGGACTTGTTCCCTGTA-3′) for *ARNT2* were designed and synthesized by Hokkaido System Science (Sapporo, Hokkaido, Japan). PCR reactions were performed on a GeneAmp® PCR System 9700 (Applied Biosystems) under the following cycling conditions: 94°C for 2 min, followed by 35 cycles of 98°C for 10 s, 60°C for 30 s and 68°C for 1 min. PCR products were then separated on 2% agarose gels containing 0.5 µl/ml ethidium bromide and imaged in a molecular imager (FX Pro Plus; Bio-Rad Laboratories, Hercules, CA, USA).

### Real-time RT-PCR

Real-time PCR was performed using TaqMan® Gene Expression Master Mix (Applied Biosystems) in accordance with the manufacturer's instructions. TaqMan® Gene Expression Assays (Applied Biosystems) used in this study were: Hs00208298_m1 for *ARNT2*, Hs00171829_m1 for *MMP11*, Hs00901551_m1 for *NTSR1*, Hs99999903_m1 for glyceraldehyde-3-phosphate dehydrogenase (*GAPDH*) and Hs99999903_m1 for beta-actin. The amplification reaction was performed in an ABI PRISM 7000 Sequence Detector (Applied Biosystems) under the following cycling conditions: 95°C for 15 min, followed by 40 cycles of 95°C for 15 s and 60°C for 60 s. Gene expression levels were calculated based on the threshold cycle using Sequence Detection System Software (Applied Biosystems). Gene expression was normalized against *GAPDH* expression and set to 1 for the control DMSO-treated cells.

### Genotyping

SNPs were determined using the GoldenGate assay (Illumina, San Diego, CA, USA) as previously reported [8].

### Statistical analysis

Quantitative data are expressed as the mean ± SEM. A non-parametric test, the Mann-Whitney U test, was applied to test for statistical significance. The effect of BPA compared with DMSO control was evaluated by a paired T-test (Wilcoxon) using R (version 2.15.0). Relationships were considered statistically significant at *P*<0.05.

## Results

### Samples

Thirty-four hFFCs derived from control children without distinct MGMs (*n* = 5; median age 5.3 yrs), HS (*n* = 21; median age 2.5 yrs) and CO (*n* = 8; median age 1.6 yrs) patients were collected. Detailed patient information is summarized in Table S1, including age, disease type and genotype of *ARNT2* SNP, *rs5000770*.

### Difference in *ARNT2* mRNA levels in hFFCs derived from CO and HS patients

**Figure 1:**
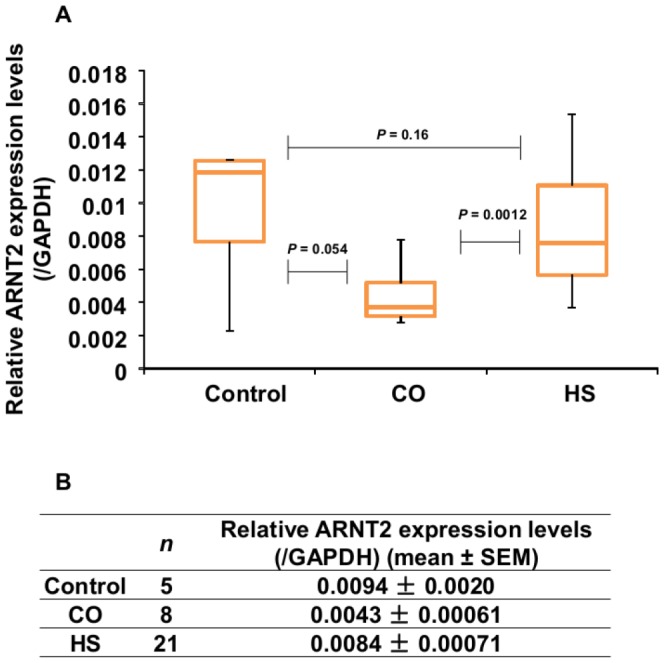
*ARNT2* mRNA levels in control children and child HS and CO patients. *ARNT2* expression were measured in hFFCs derived from the control group (*n* = 5), HS (*n* = 21) group and CO (*n* = 8) group by TaqMan® real-time PCR. (***A***) Boxplot and (***B***) summary of the quantitative data.

The mRNA levels of *ARNT2* in the control, HS and CO groups are shown in Figure 1. The mean *ARNT2* mRNA level, normalized to *GAPDH*, in the control group was 0.0094, in the HS group was 0.0084 and in the CO group was 0.0043. No significant difference was observed between the control group and HS group (*P* = 0.16), while lower *ARNT2* expression was observed in CO group compared with control group only on the borderline of significance (*P* = 0.054).

### Differences in the genetic response to BPA exposure in hFFCs derived from CO and HS patients

**Figure 2:**
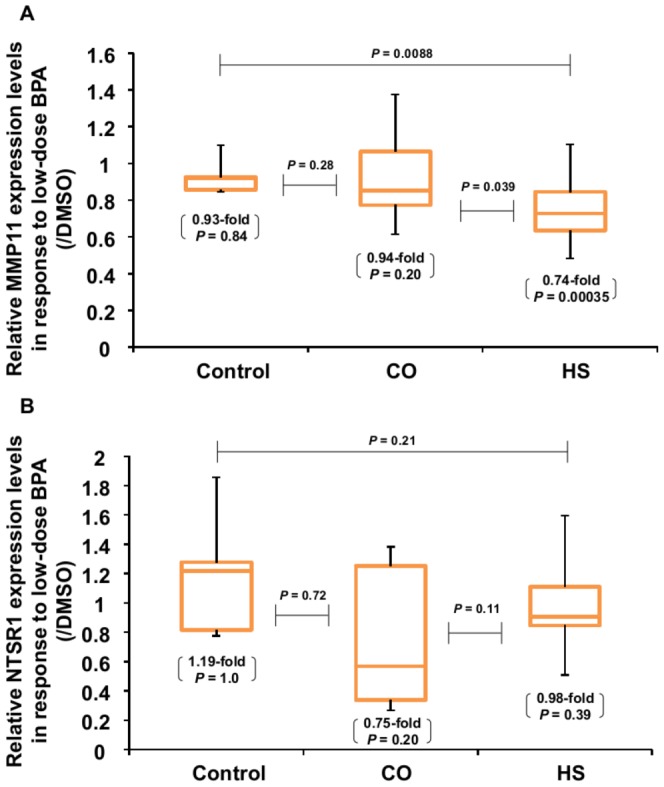
Difference in the genetic response to low-dose BPA in hFFCs derived from control children and child HS and CO patients. Cells were treated with 10 nM BPA for 24 h, and then the expression of *MMP11* (***A***) and *NTSR1* (***B***) was measured by TaqMan® real-time PCR. Significance was evaluated by the Mann-Whitney U test or Wilcoxon test. The bottom numbers indicate the fold changes and *P* value induced by BPA compared with DMSO control.

We confirmed the effect of BPA exposure on *GAPDH* expression and no effect was observed when *GAPDH* mRNA was normalized to beta-actin (Figure S2). Then, differences in hFFC *MMP11* and *NTSR1* expression in response to low-dose BPA exposure were compared among control, HS and CO groups. As shown in Figure 2A, *MMP11* expression was significantly inhibited by exposure to 10 nM BPA in the HS group (0.74-fold compared with DMSO control, *P* = 0.0035), while no significant effect was observed in the control group (0.93-fold compared with DMSO control, *P* = 0.84) and CO group (0.94-fold compared with DMSO control, *P* = 0.20). Significantly lower *MMP11* expression levels were observed in the HS group compared with the control group (0.80-fold, *P* = 0.0088) and CO group (0.79-fold, *P* = 0.039) in response to low-dose BPA exposure. As shown in Figure 2B, no significant effects in *NTSR1* expression were observed by 10 nM BPA exposure in the control group (1.19-fold compared with DMSO control, *P* = 1.0), HS group (0.98-fold compared with DMSO control, *P* = 0.39) and CO group (0.75-fold compared with DMSO control, *P* = 0.20). In addition, no significant difference in *NTSR1* expression levels was observed in the HS group or CO group compared with the control group in response to low-dose BPA exposure.

### Effect of *ARNT2* variants on *ARNT2* mRNA levels and splicing

**Figure 3:**
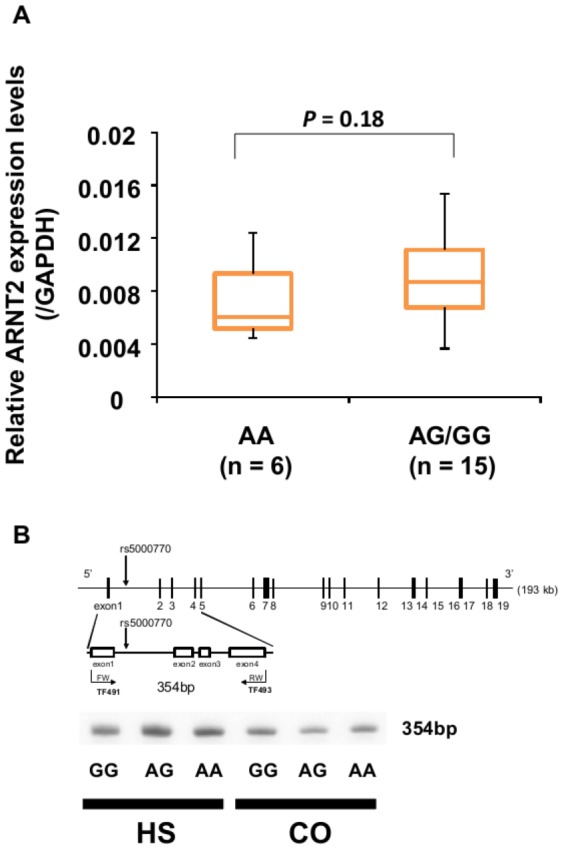
Association between *rs5000770* genotype and *ARNT2* expression. (***A***) Boxplot of the *ARNT2* mRNA level in hFFCs from child HS patients with different genotypes (6 AA and 15 AG/GG) relative to *GAPDH* measured using TaqMan® real-time PCR. Statistical significance was evaluated by the Mann-Whitney U test. (***B***) Scheme of the quantitative PCR strategy for screening splicing variants. No splicing variant was detected using intron-spanning RT-PCR in hFFCs either from HS or CO patients.

We then investigated the effect of SNP *rs5000770* genotype on *ARTN2* expression in the HS group. As shown in Figure 3A, the mean *ARNT2* mRNA levels, normalized to *GAPDH*, in the AA group were 0.0074 (*n* = 6) and in the AG/GG group, 0.0088 (*n* = 15). These results indicated no significant difference in *ARNT2* mRNA levels between the AA group and the AG/GG group (*P* = 0.18). Furthermore, as shown in Figure 3B, no significant effect of SNP *rs5000770* genotype was observed in the splicing pattern of *ARNT2* in either HS or CO groups.

### Effect of *ARNT2* variants on the genetic response to low-dose BPA exposure

**Figure 4:**
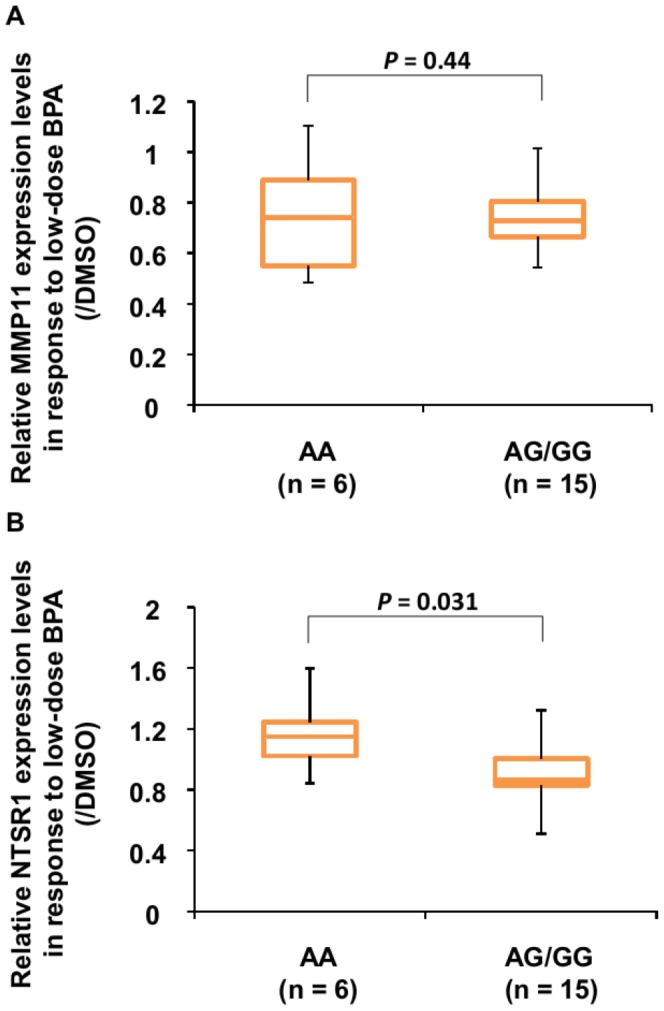
Effect of SNP *rs5000770* genotype on the genetic response to low-dose BPA. Cells were treated with 10 nM BPA for 24 h, and then *MMP11* and *NTSR1* mRNA levels were measured by TaqMan® real-time PCR. Boxplot of the quantitative data comparing *MMP11* (***A***) and *NTSR1* (***B***) expression in child HS patients with different genotypes (6 AA and 15 AG/GG). Significance was evaluated by the Mann-Whitney U test.

Finally, we investigated the effect of SNP *rs5000770* genotype on the genetic response of hFFCs to low-dose BPA exposure in the HS group. As shown in Figure 4A, no significant difference in *MMP11* expression in response to 10 nM BPA exposure was observed between the AA and AG/GG groups (*P* = 0.44). However, as shown in Figure 4B, a significant difference in *NTSR1* gene expression in response to 10 nM BPA exposure was observed between the AA and AG/GG groups (*P* = 0.031). However, no significant effects of low-dose BPA exposure were observed in *NTSR1* gene expression compared with DMSO control in either AA or AG/GG groups (1.16-fold; *P* = 0.56 and 0.90-fold; *P* = 0.19, respectively).

## Discussion

Variability in susceptibility to the effects of EEDs exposure has been considered as a contributing factor to MGMs [7]. However, to our knowledge, no previous report has tested this hypothesis. Thus, this study was initiated to investigate how the genetic response of individuals varied to low-dose BPA exposure using hFFCs derived from child CO and HS patients. Differences in *MMP11* and *NTSR1* expression in response to 10 nM BPA treatment in CO and HS groups were compared with that in control children. Furthermore, the effect of SNP *rs5000770* (G>A) genotype on individual sensitivity to low-dose BPA was also investigated in the HS group. The concentration of BPA used in this study was 10 nM, which is below the dose of 50 µg/kg/day (approximately 200 nM for *in vitro* cell or organ culture studies) usually considered as safe for humans and in the concentration range of 1–19.4 nM that is commonly detected in human tissues and fluids [15], [21].

One of our most interesting results is the specificity of the effect of low-dose BPA on *MMP11* expression. Despite increasing evidence that exposure to BPA causes adverse health effects in humans, controversy remains about the specificity of these effects [6]. In this study, we observed that 10 nM BPA exposure can significantly inhibit *MMP11* expression in hFFCs derived from child HS but not control children and CO patients. We have recently identified that *MMP11* is a novel target of low dose of BPA exposure and that BPA can inhibit *MMP11* expression in a dose-dependent manner in hFFCs derived from child HS patients. Furthermore, we found that mRNA levels of *MMP11* were significantly lower in the HS group compared with the control and CO groups suggesting that the involvement of BPA in the development of HS might be related with the downregulation of *MMP11* [17]. The results of this study are in accord with this hypothesis. MMPs are known to be involved in the breakdown of extracellular matrix in normal physiological processes, such as embryonic development, reproduction, and tissue remodeling [22], [23]. Knockdown of MMPs in *Tribolium* using genetic interference has been related with malformation in tracheal and gut development during beetle embryogenesis and pupal morphogenesis [24]. It is known that epithelial seam formation and remodeling during urethral formation play important roles in the etiology of HS. The urethral abnormalities seen in HS can be viewed as a failure of epithelial cell adhesion [25]. Therefore, if our hypothesis that downregulation of *MMP11* plays an important role in the etiology of HS is correct, it is important to note that the outcome of continuous low-dose BPA exposure with respect to the risk for HS development might be influenced by individual variations of *MMP11* expression in response to low-dose BPA.

Another interesting finding of our study is that *ARNT2* mRNA levels are lower in the HS and CO groups compared with the control group. However, no significant differences were observed (*P* = 0.16 and 0.054, respectively). Compared with its homolog *ARNT*, *ARNT2* has a more restricted pattern of expression, commonly found in the central nervous system and other developing organs, such as the kidney [26]. The main function of *ARNT2* seems to be in organ development, since *Arnt2* knockout mice and zebrafish suffer severe developmental defects and die shortly after birth [13]. Our study suggests limited evidence that inhibition of *ARNT2* expression might be involved in the development of HS and CO. It is well known that *ARNT2* acts as a common obligate partner for several members of the bHLH-PAS family, including aryl hydrocarbon receptor (AHR) and hypoxia-inducible factor (HIF)-1α [12]. Our previous study has reported that downregulation of *ARNT2* expression can affect HIF signaling and metabolism in human breast cancer cells [27], [28]. HIF is well-known to play a key role in many developmental pathways. Further study to elucidate the role of dysregulated HIF signaling during genital tubercle development might increase our knowledge of the etiology of HS and CO.

We did not identify *NTSR1* as a target of low-dose BPA in our previous genome-wide screen, probably owing to a lack of statistical significance. This may indicate that variability in *NTSR1* gene expression among individuals might exist in response to low-dose BPA exposure [17]. This is in accord with our present findings that no significant effects to *NTSR1* gene expression in response to low-dose BPA exposure were observed in all the three groups (Figure 2B). However, we found that *NTSR1* expression in response to low-dose BPA was significantly different between the SNP *rs5000770* AA group and the AG/GG group in child HS patients (*P* = 0.031). It has been reported that *NTSR1* activation leads to cell proliferation, survival, mobility, and invasiveness in specific cancer cell types [29]. Furthermore, the transcription of neurotensin, mainly mediated by *NTSR1*, is enhanced by estradiol in human breast epithelial cells [20]. Of interest, *NTSR1* expression in SNP *rs5000770* AA carriers tends to be increased in response to low-dose BPA (1.16-fold compared with DMSO control) while in AG/GG carriers tends to be decreased (0.90-fold compared with DMSO control). One of the likely explanations for these findings is that SNP *rs5000770* AA carriers might be more sensitive to the estrogen-like effect of low-dose BPA. However, we found no difference in *ARNT2* expression or in *ARNT2* splicing variants in mRNA obtained from hFFCs of child HS and CO patients of each SNP *rs5000770* genotype. And also, it should be noted that no significant effects of low-dose BPA exposure were observed in *NTSR1* gene expression compared with DMSO control in either AA or AG/GG groups. Further analyses will be needed to demonstrate the underlying mechanism by which this SNP might influence the inter-individual variations in sensitivity to the effect of low-dose BPA and increased risk of HS. It is known that *ARNT2* is located on 15q24-25, while 15q24 microdeletion syndrome has been recently described as a recurrent, submicroscopic genomic imbalance found in individuals with developmental delay, craniofacial dysmorphism, digital and genital abnormalities (including HS) [30], [31], [32]. Other possible interpretations of our findings include the interactions between genetic loci located on 15q24-25, although further study is necessary to investigate this possibility.

In summary, we observed the specific effect of low-dose BPA inhibiting *MMP11* expression in child HS but not control children and CO patients. Furthermore, we observed a significant difference in *NTSR1* expression in response to low-dose BPA among HS patients with different SNP *rs5000770* genotypes, suggesting that variability in genetic susceptibility to the effects of EEDs exposure might contribute to HS.

## Supporting Information

Figure S1 **Expression of top genes with the highest standard deviations in response to low-dose BPA.** Gene expression profiles of three hFFCs derived from HS patients were measured after exposure to 10 nM BPA for 24 h. Differentially expressed genes were listed according their standard deviations among the three cell lines. Data derived from our previous microarray study (Qin *et al.* PLoS One. 2012;7(5):e36711).
(PPTX)

Figure S2 **Effect of BPA exposure on** ***GAPDH*** **expression.** (***A***) *MMP11* and (***B***) *GAPDH* expression following BPA treatment were measured in hFFCs derived from a control child without MGMs. Gene expression was normalized against *GAPDH* or beta-actin expression and set to 1 for the control DMSO-treated cells.
(PPTX)

Table S1 **Summary of patient characteristics.**
(DOCX)
