# Supplementary material for: Individual Variation of the Genetic Response to Bisphenol A in Human Foreskin Fibroblast Cells Derived from Cryptorchidism and Hypospadias Patients
Source: PLoS One. 2012 Dec 28;7(12):e52756. doi: 10.1371/journal.pone.0052756 (PMC3532342; doi:10.1371/journal.pone.0052756)
Supplement: Table S1 — Summary of patient characteristics. (DOCX) [file pone.0052756.s003.docx]

**Table S1. Summary of patient’s characteristics**

| ID | Age (year) | Disease | Type | Genotype of rs5000770 |
| --- | --- | --- | --- | --- |
| 1 | 1.9 | CO | both inguinal | AG |
| 2 | 1.3 | CO | right: retractile testis; left: abdominal | AG |
| 3 | 5.9 | CO | right: inguinal; left: retractile testis | AA |
| 4 | 1.0 | CO | right: normal; left: abdominal | AG |
| 5 | 2.2 | CO | both gliding testis | AA |
| 6 | 1.1 | CO | right: normal;left: inguinal | GG |
| 7 | 2.5 | CO | right: normal;left: inguinal | AA |
| 8 | 1.5 | CO | right: inguinal;left: normal | AA |
| 9 | 1.1 | HS | midshaft | GG |
| 10 | 1.7 | HS | proximal | AG |
| 11 | 1.6 | HS | proximal | AG |
| 12 | 13.3 | HS | distal | AG |
| 13 | 2.6 | HS | distal | AG |
| 14 | 1.9 | HS | distal | GG |
| 15 | 1.7 | HS | proximal | AG |
| 16 | 1 | HS | proximal | AG |
| 17 | 1.8 | HS | proximal | AA |
| 18 | 1.2 | HS | proximal | AG |
| 19 | 5.4 | HS | glans | GG |
| 20 | 3.7 | HS | penile | GG |
| 21 | 0.8 | HS | proximal | GG |
| 22 | 0.8 | HS | penile | AA |
| 23 | 1.2 | HS | penile | AA |
| 24 | 1 | HS | proximal | AA |
| 25 | 1.2 | HS | perineal | AG |
| 26 | 2.2 | HS | scrotal | AG |
| 27 | 1.0 | HS | proximal | AA |
| 28 | 0.9 | HS | proximal | AG |
| 29 | 2.8 | HS | proximal | AA |
